# Effect of Annealing on LiCoO_2_ Thin Film Deposited by RF Magnetron Sputtering

**DOI:** 10.3390/ma18061217

**Published:** 2025-03-09

**Authors:** Zohra Benzarti, José David Castro, Edgar Carneiro, Lara Pacheco, Nelson Duarte, Sandra Carvalho, Ricardo Serra, Albano Cavaleiro, Cristiana Alves, Sandra Cruz

**Affiliations:** 1 CEMMPRE, ARISE, Mechanical Engineering Department, University of Coimbra, 3030-788 Coimbra, Portugal; jodcastroca@unal.edu.co (J.D.C.); lb.pacheco@campus.fct.unl.pt (L.P.); sandra.carvalho@dem.uc.pt (S.C.); ricardo.serra@dem.uc.pt (R.S.); albano.cavaleiro@dem.uc.pt (A.C.); sandracruz@ipn.pt (S.C.); 2 Laboratory of Multifunctional Materials and Applications (LaMMA, LR16ES18), Faculty of Sciences of Sfax, University of Sfax, B.P. 1171, Sfax 3000, Tunisia; 3 CF-UM-UP, Centro de Física das Universidades do Minho e do Porto, University of Minho, Campus of Azurém, 4800-058 Guimarães, Portugal; eadgarcarneiro@gmail.com; 4 IPN—LED & MAT—Instituto Pedro Nunes, Laboratory of Tests, Wear and Materials, Rua Pedro Nunes, 3030-199 Coimbra, Portugal; nelson.duarte@ipn.pt; 5 INL—Laboratório Ibérico Internacional de Nanotecnologia, Av. Mestre José Veiga s/n, 4715-330 Braga, Portugal; cristiana.alves@inl.int

**Keywords:** LiCoO_2_ cathode, sputter deposition, microstructural and electrochemical properties, Li-ion batteries

## Abstract

This study investigates the properties of LiCoO_2_ coatings as cathodes for lithium-ion batteries, focusing on the effects of annealing on their structural, morphological, chemical, vibrational, and electrochemical characteristics. The LiCoO_2_ coatings were deposited on silicon and glass substrates using RF magnetron sputtering at 100 W and subsequently annealed at 600 °C for 1 h. The films were characterized before and after annealing using X-ray diffraction (XRD), scanning electron microscopy (SEM), energy-dispersive spectroscopy (EDS), X-ray photoelectron spectroscopy (XPS), Raman spectroscopy, and electrochemical impedance spectroscopy (EIS). Annealing improved the crystallinity of LiCoO_2_, which is critical for enhancing lithium-ion diffusion. Furthermore, an XPS analysis revealed a layered structure with a Li-rich outer layer and a Co-rich underlayer, indicating a more uniform distribution of Li and Co, along with increased oxygen content. Additionally, the annealing process refined the microstructure of the LiCoO_2_ coating, positively impacting its electrochemical performance. A comparative analysis of cyclic voltammetry (CV) and galvanostatic charge/discharge (GCD) results demonstrated a significant improvement in the charge/discharge capacity post-annealing. This study successfully highlights the beneficial effects of annealing on LiCoO_2_ thin-film cathodes, offering valuable insights for developing more efficient and sustainable lithium-ion batteries through sputter-deposition processes.

## 1. Introduction

The major challenges of the 21st century, including global climate change and the depletion of fossil energy reserves, have driven the demand for clean and sustainable energy solutions. Among these, rechargeable batteries are essential for energy storage, requiring high energy retention over extended periods, economic viability, and minimal environmental impact. Lithium-ion batteries (LIBs) have emerged as a leading solution, dominating the rechargeable battery market due to their high energy density, long lifespan, and absence of memory effect [1,2]. These features make LIBs particularly suitable for applications where space and weight are critical, such as portable electronics and electric vehicles.

The performance of LIBs depends heavily on the cathode material, which accounts for over 40% of the total battery weight and plays a pivotal role in determining capacity and energy density. LiCoO_2_, introduced in 1980 as the first cathode material, remains a benchmark due to its high capacity, energy density, and stability [3]. However, delithiating LiCoO_2_ by more than 50% causes irreversible oxidation and structural collapse due to oxygen release, limiting its performance [4]. To address this, increasing the charging voltage has been proposed as a promising strategy to enhance energy density [4]. The layered oxide structure of LiCoO_2_ facilitates lithium-ion migration more effectively than other lithium metal oxide structures, contributing to its high specific energy density, low self-discharge, and excellent cycle life [1]. Despite these advantages, achieving a pure crystalline phase of LiCoO_2_ films remains a challenge, and clear experimental protocols for optimizing these films are still lacking. Therefore, obtaining LiCoO_2_ thin films with the desired crystal structure is essential to fully exploit their advantages, including high electronic conductivity and capacity. Given LiCoO_2_’s anisotropic nature, grain-oriented and high specific surface planes are desirable to enhance lithium diffusion at the electrode–electrolyte interface and stabilize the rhombohedral crystal phase [5].

Thin-film LIBs offer significant advantages by eliminating inactive binders, reducing weight, and increasing energy capacity. Indeed, several strategies have been explored, including optimizing film thickness to balance mechanical stability and electrochemical performance [6], enhancing electrode porosity to improve electrolyte penetration and lithium-ion transport [7], and incorporating nanostructured architectures to increase the effective surface area and active sites for ion intercalation [8]. Additionally, improving ionic and electronic conductivity through doping [4] or multilayered designs [9] can enhance overall energy efficiency and capacity retention. These approaches aim to maximize the electrochemical performance of thin-film LiCoO_2_ cathodes while maintaining their advantages in miniaturized energy storage applications.

Physical vapor deposition (PVD) techniques, such as magnetron sputtering, enable the production of thin-film electrodes with lower internal resistance and high-rate cyclability due to their compact and void-free structure [9]. Among the PVD techniques, RF magnetron sputtering stands out from other methodologies [10], particularly for its ability to deposit insulating materials, its scalability, and its high reproducibility [11,12]. The power applied during RF magnetron sputtering depends heavily on system-specific factors such as the cathode size, cooling water configuration, and chamber dimensions, which can significantly vary across instruments and affect deposition outcomes [13]. This variability underscores the need for tailored optimization in each setup. Indeed, the working power during sputtering is a critical parameter as it controls the size of particles deposited from the target material, affecting the structural, morphological, and chemical properties of LiCoO_2_ coatings [6,14]. For instance, Liao et al. [15] demonstrated that the Li/Co ratio in sputtered films varies with RF sputtering power due to differences in atomic weights. At 100 W, the Li/Co ratio approaches 1, which is optimal for achieving high Li^+^ ion mobility and enhanced electrochemical performance [15]. Additionally, Alkan et al. [16] employed RF magnetron sputtering for the deposition of LiCoO_2_ thin films, gradually increasing the applied power from 0 W to 125 W at a rate of 2 W/min to prevent target degradation, which could result in cracking, material loss, and reduced film quality.

Post-deposition annealing is essential for transforming amorphous LiCoO_2_ films into crystalline structures, improving lithium-ion diffusion, and stabilizing the rhombohedral crystal phase [12,17]. Annealing temperatures above 550 °C induce a preferred orientation of the (101) and (104) planes, creating efficient pathways for Li^+^ ion diffusion [18]. Insufficient heating (below 400 °C) results in poor crystallization, while excessive temperatures (e.g., 650 °C) can cause lithium evaporation and grain distortion, leading to a Li^+^ deficiency and diminishing performance [18].

This study employed a comprehensive suite of advanced characterization techniques to investigate the effects of annealing on RF-magnetron-sputtered LiCoO_2_ thin films. The films were deposited at 100 W to ensure precise control over the composition [19]. The novelty of this work lies in its multi-technique approach to understanding how annealing influences the structural, chemical, morphological, and electrochemical properties of LiCoO_2_ thin films, providing essential insights for optimizing their performance in LIBs. This systematic investigation of annealing effects on RF-sputtered LiCoO_2_ thin films bridges gaps in understanding the interplay between processing conditions, structural evolution, and electrochemical performance, offering an experimental protocol for engineering high-capacity cathodes to develop high energy density and durable energy storage systems.

## 2. Materials and Methods

### 2.1. Material Deposition

LiCoO_2_ coatings were deposited at room temperature on silicon substrates (type 100 P) and commercial glass (Normax—76 × 26 × 1 mm). The glass substrates were used for electrochemical tests to isolate the coating’s response and minimize the influence of the substrate material. Before depositions, all substrate materials were cleaned with water, acetone, and ethanol using an ultrasonic bath for 10 min in each medium and then dried with a hot air dryer. The LiCoO_2_ coating was deposited by RF magnetron sputtering in a home-made vacuum chamber. The argon flow was maintained at a constant 80 sccm. The power applied to the LiCoO_2_ ceramic target (200 × 100 × 6 mm^3^, with a copper backing; Testbourne Ltd. (Basingstoke, Hampshire, United Kingdom) connected to the RF source was maintained at 100 W. Due to the oxygen-rich nature of the LiCoO_2_ target, achieving an adequate base pressure of approximately 1 × 10^−6^ mbar was the priority in each deposition to reduce the reactive atoms inside the chamber. The working pressure was maintained at around 4 × 10^−3^ mbar. The distance between the LiCoO_2_ target and the substrate holder was about 10 cm, and the substrate holder rotation speed was set at 7 rpm. The LiCoO_2_ deposition time was 14,400 s, resulting in a thickness of about 250 nm.

### 2.2. Material Characterization

The structural properties of the coating were studied using X-ray diffraction (XRD). A Philips X’ Pert Pro diffractometer (Co radiation; PANalytical, Almelo, Netherlands) was used. High-temperature experiments were carried out in a vacuum atmosphere (with a flux of Ar + 5% H_2_) using an Anton Paar high-temperature attachment (HTK-16; Graz, Austria). The sample was mounted on a platinum strip, which served as both the sample holder and the heating element (Pt/Pt–Rh thermocouple). Data were collected in continuous mode using grazing incidence X-Ray diffraction (GIXRD), with an incidence angle of 2° over a 2θ range of 10–70°, a step of 0.025°, and at a rate of 2°/min. Measurements were performed at temperatures ranging from room temperature to 600 °C. The sample was heated at a rate of 5 °C/min, with a 60 min stabilization period before each scan.

Vibrational modes of the thin film were analyzed using Raman spectroscopy with a Renishaw in Via™ confocal Raman microscope (Wotton-under-Edge, United Kingdom), employing a laser excitation wavelength of 532 nm in the frequency range of 200–1200 cm^−1^.

Surface morphology and coating thickness were examined using scanning electron microscopy (SEM) (Zeiss-Merlin field emission, Oberkochen, Germany), operating at 2 keV and equipped with energy-dispersive spectroscopy (EDS) for the chemical composition analysis. The EDS operating conditions were set at an accelerating voltage of 10 kV, a current of 1 nA, and a magnification of 1000×. The analyzed area was approximately 114 µm × 85 µm (9690 µm^2^), with three areas examined per sample. X-ray photoelectron spectroscopy (XPS—Thermo Scientific (Waltham, MA, USA) Escalab 250 Xi) spectra were obtained using non-monochromatic Al Kα radiation under a base pressure below 5 × 10^−10^ mbar. Different settings were used for the survey spectra (150 eV pass energy; 1.0 eV energy step size) and the high-resolution spectra (40 eV pass energy; 0.1 eV energy step size). Samples were etched using a monoatomic Ar^+^ ion beam with a kinematic energy of 1 keV and a current of 3 μA until a depth of approximately 191 nm was reached. The resulting spectra were post-processed using CasaXPS 2.3.18PR1.0 software, and the electric charge effect was corrected by setting the main C 1s peak component at 285.0 eV.

To characterize the electrochemical properties of the LiCoO_2_ coating, a glass cell (Gamry instruments; lithium battery cell) connected to a GAMRY Interface 5000 E potentiostat was used. The coating served as the working electrode, while Li foil was used as both the reference and counter electrodes. The electrolyte was a mixture of lithium perchlorate (LiClO_4_) and ethylene carbonate (EC) with dimethyl carbonate (DEC) solvents (1 M LiClO_4_ in 1:1 (*v*/*v*) EC/DEC). All cells were sealed in an argon-filled glovebox (PLAS-LABS, Lansing, MI, USA) to prevent air exposure, protecting the reactive lithium foil from degrading, the sensitive electrolyte from forming destabilizing byproducts, and the LiCoO_2_ coating from unwanted surface reactions. After sealing, the cell was placed in a Faraday cage to block electromagnetic interference, ensuring accurate data collection. Cyclic voltammetry (CV) was performed on the sample by applying a potential sweep at a rate of 1 mV/s between 0.0 and 5.0 V. Galvanostatic charge/discharge (GCD) tests were conducted by charging LiCoO_2_ to 3.0 V then discharging it to 0.7 V at a rate of 0.85 C, calculated from the specific capacity of approximately 232 mAh/g determined in preliminary tests. All cells were cycled at room temperature in the full-cell configuration. Additionally, GCD testing was performed on the annealed sample, which involved increasing the charging potential to 4.5 V.

## 3. Results and Discussion

This work aimed to obtain a LiCoO_2_ coating with optimized electrochemical properties by investigating the impact of the annealing process on its physical and chemical characteristics. The LiCoO_2_ coating was characterized both before and after annealing, focusing on its morphological, chemical, structural, and electrochemical properties. The primary objective was to evaluate how the annealing process influenced the performance and efficiency of the battery.

### 3.1. Morphological, Structural, and Chemical Aspects of LiCoO_2_

#### 3.1.1. SEM and EDS Analyses

**Figure 1:**
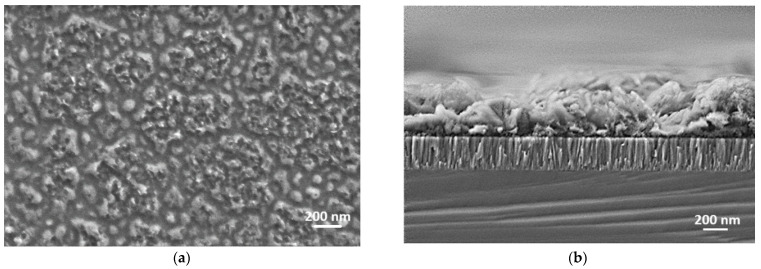
Surface morphology (**a**) and SEM cross-sectional images (**b**) of as-deposited LiCoO_2_ coating on silicon substrate.

**Figure 2:**
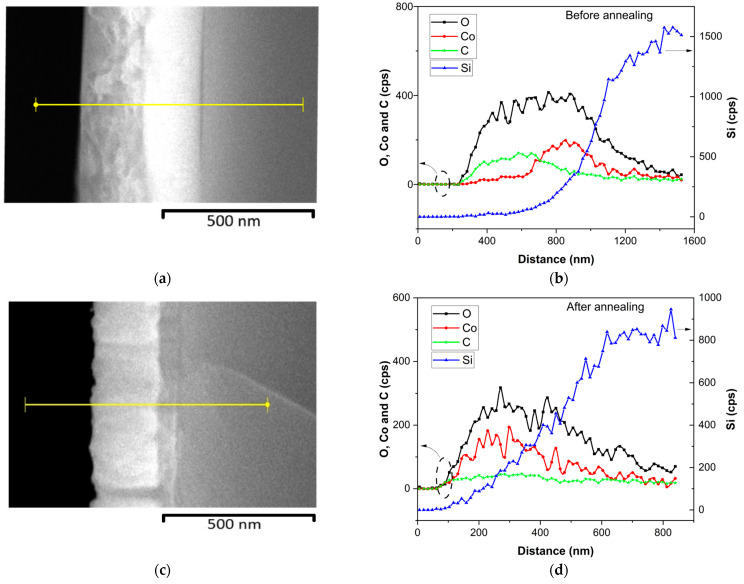
LiCoO_2_ coating: (**a**) SEM micrograph before annealing, (**b**) EDS line profile before annealing, (**c**) SEM micrograph after annealing at 600 °C, and (**d**) EDS line profile after annealing at 600 °C.

Figure 1 presents the surface morphology and SEM cross-sectional images of the as-deposited LiCoO_2_ coating. The surface morphology revealed the formation of islands on the coating’s surface, while the cross-sectional SEM image exhibited the following two layers: a top layer likely corresponding with the surface islands and a bottom layer with a columnar structure. Additionally, the EDS analysis showed a higher oxygen content (55%) compared with cobalt (12%). The SEM cross-sectional image also highlighted the presence of an additional thin top layer, likely formed due to exposure to air. To further investigate the chemical composition of this top layer, an EDS line profile of the LiCoO_2_ coating was produced, both before and after annealing at 600 °C (Figure 2). Notably, in the top layer before annealing, cobalt (Co) was nearly absent, while oxygen (O) and carbon (C) were present in significant amounts, as shown in Figure 2b. This top layer was likely composed of Li_2_CO_3_ [20], which may have formed during exposure to air containing trace amounts of CO_2_. After annealing, this top layer disappeared (Figure 2c), revealing a single crystallized layer consisting of Co, O, and likely Li elements, as illustrated in Figure 2d.

To elucidate the growth morphology observed in the cross-sectional SEM images, the Thornton structure zone model (SZM) for sputtered coatings could be applied, using the homologous temperature ratio (Ts/Tm), where Ts is the substrate temperature and Tm is the melting temperature of LiCoO_2_, approximated to be 1300 °C (1573 K) [21]. During RF magnetron sputtering, deposition occurred at room temperature (~25 °C or 298 K), yielding a Ts/Tm of ~0.19. This low ratio placed the as-deposited film in Zone 1 of Thornton’s SZM, where limited adatom mobility leads to a columnar structure with voids due to self-shadowing and ballistic deposition, as observed in the bottom layer. The island-like top layer likely resulted from post-deposition reactions (e.g., Li_2_CO_3_ formation, confirmed by EDS and XPS; Section 3.1.1 and Section 3.1.4). Post annealing at 600 °C (873 K), Ts/Tm increased to ~0.55, shifting the morphology toward Zone 2, where enhanced adatom diffusion promoted recrystallization and densification, eliminating the top layer and refining the structure, consistent with improved crystallinity (Section 3.1.2) and electrochemical performance (Section 3.2).

#### 3.1.2. XRD Analysis

The behavior of materials, including thin films, changes with their properties. It is, therefore, imperative to understand the chemical and structural changes induced by treatments such as annealing that promote these modifications.

In situ high-temperature XRD (HTXRD) enables the in-house XRD analysis of materials at variable temperatures and under different atmospheric conditions. XRD analysis was used to determine the crystallinity of the deposited LiCoO_2_ thin film, with diffraction patterns measured accordingly. Li-ion diffusion favors perpendicular movement on the substrate surface through the film thickness, with (101) and (104) plane orientations preferred as opposed to the (003) plane orientation, which impedes such transport [22,23]. This aligns with the functional requirement of thin-film cathodes for efficient diffusion.

**Figure 3:**
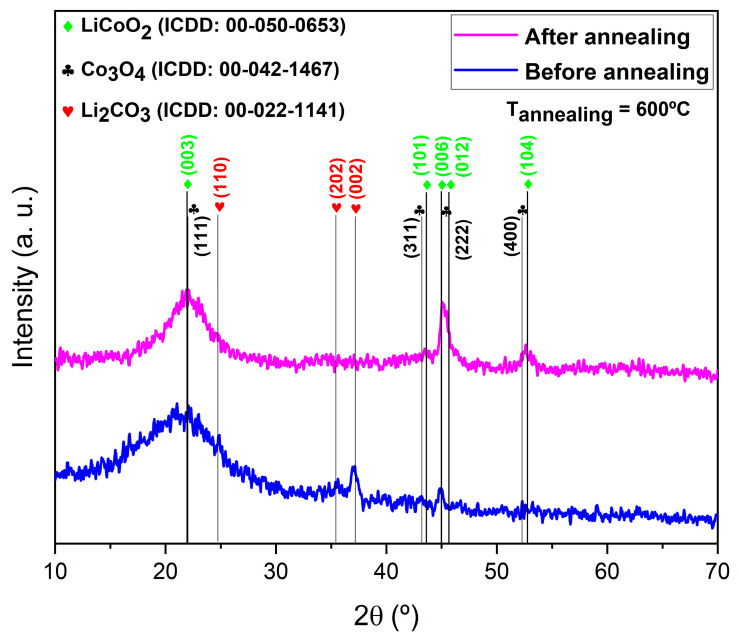
XRD patterns of the LiCoO_2_ thin films before and after annealing, scanned in a vacuum at room temperature and at 600 °C.

Initially, the sample required annealing to achieve optimal crystallinity, as it was somewhat amorphous in its as-deposited state. To address this, the sample was annealed from room temperature (approximately 25 °C) to 600 °C. Figure 3 shows the XRD patterns of the LiCoO_2_ coating before and after annealing. Annealing was conducted in an Ar gas atmosphere, with a temperature ramp rate of 5 °C/min. The increase in annealing temperature improved the structural properties, as evidenced by the narrowing of the diffraction peaks. Additionally, the diffraction peaks associated with the Li_2_CO_3_ phase, particularly the (002) plane at 37.09°, disappeared entirely [20], consistent with the EDS findings.

The (101) and (006) planes of the LiCoO_2_ coating, observed at 43.78° and 44.90°, respectively, intensified after annealing at 600 °C. Mendoza et al. [24] reported that the LiCoO_2_ crystal structure is strongly influenced by the presence of broad or well-resolved peaks corresponding with the (006) and (012) planes at 44.90° and 45.75°, respectively. Comparing the two diffractograms, the LiCoO_2_ (003) plane observed at 22.04° narrowed after annealing at 600 °C. Moreover, the LiCoO_2_ (104) plane, observed at 53.10° and known to favor improved Li-ion diffusion, confirmed the significant role of annealing in enhancing the structural properties of the LiCoO_2_ coating.

To quantify the improvement in LiCoO_2_ coating crystallinity due to the annealing process, crystallite sizes were calculated using the Scherrer equation. Before annealing, the (003) peak at 22.04° exhibited an estimated full width at half maximum (FWHM) of approximately 0.4°, corresponding with a crystallite size of about 23.5 nm. After annealing at 600 °C, the FWHM decreased to approximately 0.25°, leading to an increased crystallite size of approximately 37.6 nm, indicating a roughly 60% improvement. This confirmed enhanced crystallinity, consistent with the intensified (101) and (006) peaks observed in Figure 3.

#### 3.1.3. Raman Analysis

**Figure 4:**
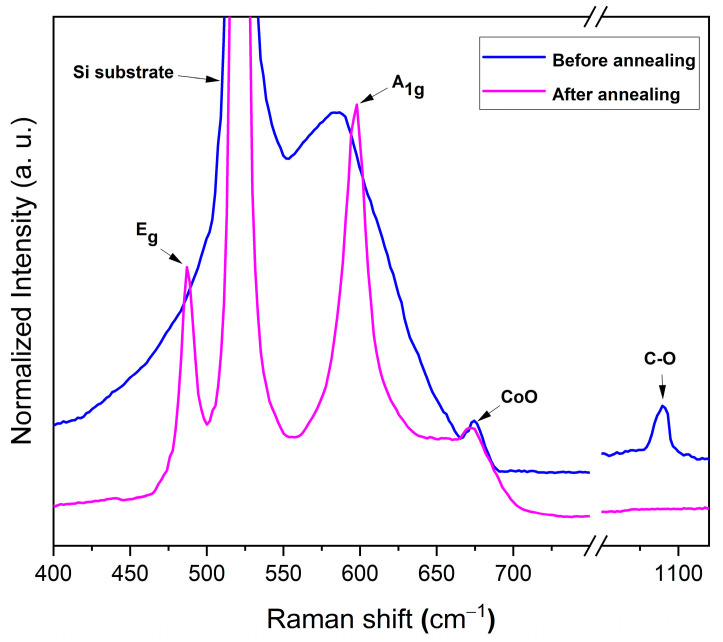
Room-temperature Raman spectra of LiCoO_2_ coating before and after an annealing process at 600 °C.

A LiCoO_2_ coating exhibits the following two Raman-active vibrational modes: A_1g_ and E_g_. These modes primarily correspond with the vibrations of oxygen atoms. In the A_1g_ mode, the two oxygen atoms vibrate in opposite directions along the C-axis of LiCoO_2_. Conversely, the E_g_ mode involves alternating opposite-direction vibrations of oxygen atoms within the planes containing Li and Co ions [25]. Figure 4 shows the room-temperature Raman spectra of the LiCoO_2_ coating measured before and after annealing at 600 °C. Before annealing, the bands were broad, likely due to the overlap of the two overlapping bands with a silicon peak. These bands included the E_g_ (~470 cm^−1^) and A_1g_ (~585 cm^−1^) modes, consistent with theoretical predictions for a spinel structure (Fd3m) [15,26]. Additionally, a small peak at around 675 cm^−1^ corresponded with the CoO cubic phase [27], while a peak at 1089.6 cm^−1^ was attributed to the symmetric stretching of C–O bonds in the Li_2_CO_3_ phase [20]. After annealing, only the two main peaks of LiCoO_2_, E_g_ and A_1g_, were observed at 486 cm^−1^ and 595 cm^−1^, respectively. These findings aligned with the results reported by Hwang and Fresh [26], Mendoza et al. [24], and Kumar et al. [28]. Notably, a blue shift of the E_g_ and A_1g_ peaks occurred after the annealing, as previously reported by Pan and Yang [6]. It is worth noting that the CoO peak persisted after annealing, unlike the C−O peak, which disappeared, confirming the removal of the Li_2_CO_3_ top layer during annealing. This observation was consistent with the EDS and XRD findings. Specifically, the XRD and EDS analyses of the LiCoO_2_ coating at room temperature revealed the presence of the Li_2_CO_3_ phase, which vanished after annealing.

#### 3.1.4. XPS Analysis

**Figure 5:**
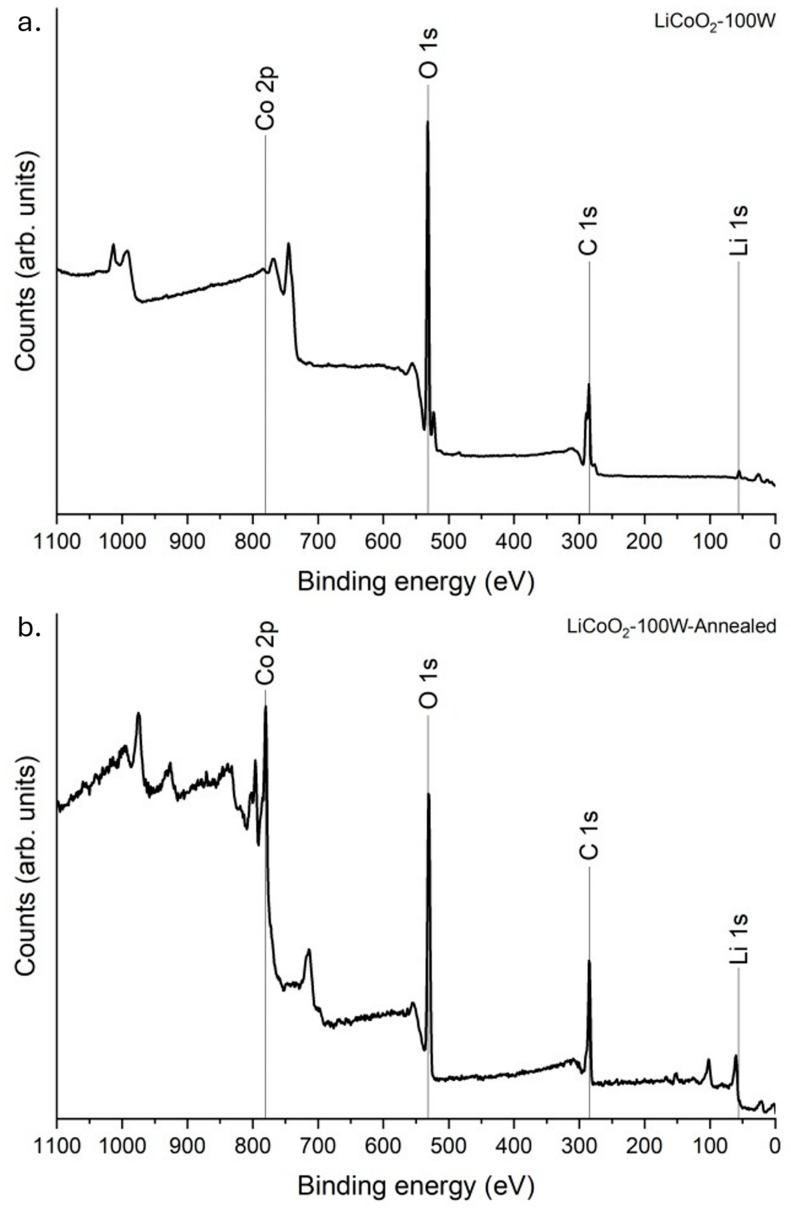
XPS survey spectra of LiCoO_2_ coating before (**a**) and after (**b**) annealing. The spectra were obtained without any etching process.

To investigate the chemical bonding in the LiCoO_2_ coating before and after annealing, XPS measurements were conducted. Figure 5 shows certain distinct differences between the two samples. The main effect of annealing on the superficial bonding of LiCoO_2_ was the detection of cobalt. As shown in Figure 2, this element was in the underneath layer of the untreated coating. This observation is discussed later, but it was evident that the exterior layer of the as-deposited coating was Li-rich, as discussed earlier. After annealing, both Co and Li were detected at the sample surface, suggesting a redistribution of these elements within the coating.

**Figure 6:**
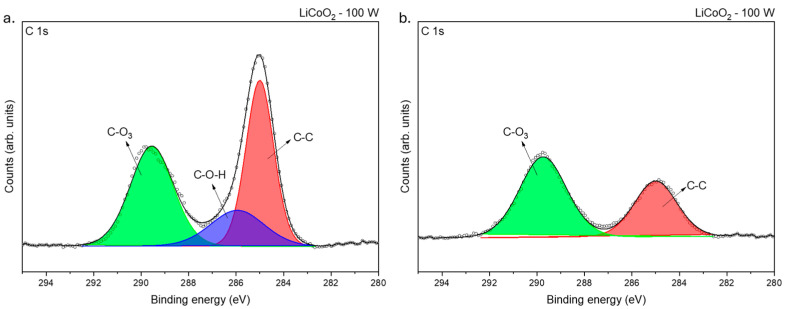
XPS spectra of LiCoO_2_ coatings before (**a**) and after (**b**) 200 s of Ar^+^ bombardment at a C 1s binding level.

Based on the composition of the sample, XPS high-resolution spectra were taken in the following regions of interest (ROIs): Li 1s, C 1s, O 1s, and Co 2p. The binding energy peaks detected in the samples before and after etching are summarized in Table 1. Figure 6 shows the ROI spectra at the C 1s binding level before and after etching for the untreated LiCoO_2_ coating. Regardless of the heat treatment and the etching process, the C 1s spectra were similar in both samples. Two different peaks were detected at ~285.94 and ~289.83 eV, which corresponded with C−O−H and C−O_3_ bindings [29]. It is remarkable that after etching, the C−O−H bond disappeared and the C−O_3_ bond remained. This bonding could indicate the easiness of LiCoO_2_ to react with C and H after being exposed for a short time to ambient conditions [30,31]. Compared with other materials, it is common that the intensity of the C−C bond peak, used to adjust the XPS spectra, decreases after etching and the other carbon chemical bonds are not detected. However, it was not the case here; therefore, it implied that C actively reacted with LiCoO_2_. This point is discussed later.

**Figure 7:**
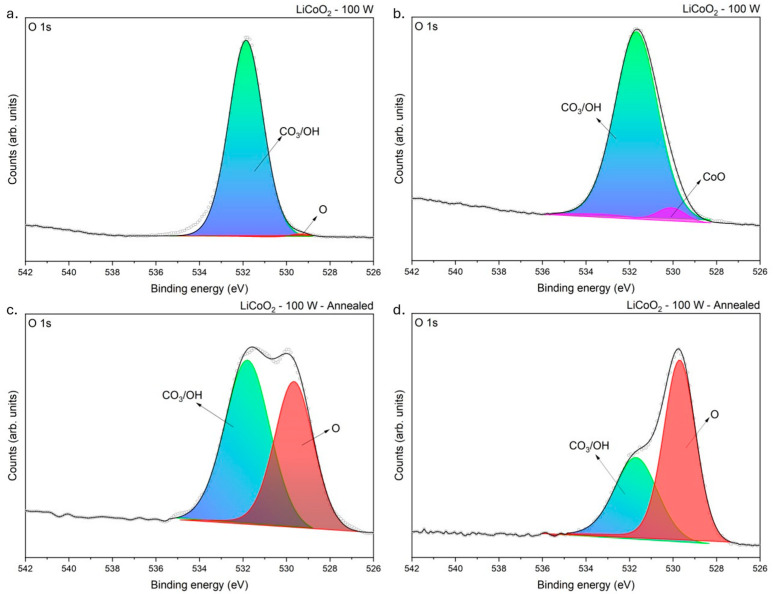
XPS spectra of LiCoO_2_ coatings before (**a**,**c**) and after (**b**,**d**) 200 s of Ar^+^ bombardment at an O 1s binding level.

Figure 7 shows the O 1s spectra from LiCoO_2_ coatings before and after annealing. As established in Figure 6, C−O−H and C−O_3_ bonds were present in the coating. At the O 1s core level, a unique peak was detected at ~531.73 eV, consistent with the mentioned chemical bonds, and labelled CO_3_/OH [29,32,33]. Figure 7 reveals the persistent presence of this bond regardless of the sample state, suggesting that ambient air, which reacted with the coating, was likely the source of the carbonate $({CO}_{3}^{2-}$) and hydroxyl (${OH}^{-}$) compounds. Additionally, an O bond was detected at ~529.67 eV, consistent with inherent oxides in the coating. Even though this bond’s signal was weak in the untreated sample, it became more pronounced after annealing, indicating that annealing enhanced the oxidation of the coating. Up to this point, identifying the exact component (i.e., oxides or carbonates) formed in the coating had remained challenging due to the presence of Li and Co. However, after etching the untreated LiCoO_2_ coating, a slight peak at 530.26 eV, consistent with CoO, was detected (Figure 7b) [34].

**Figure 8:**
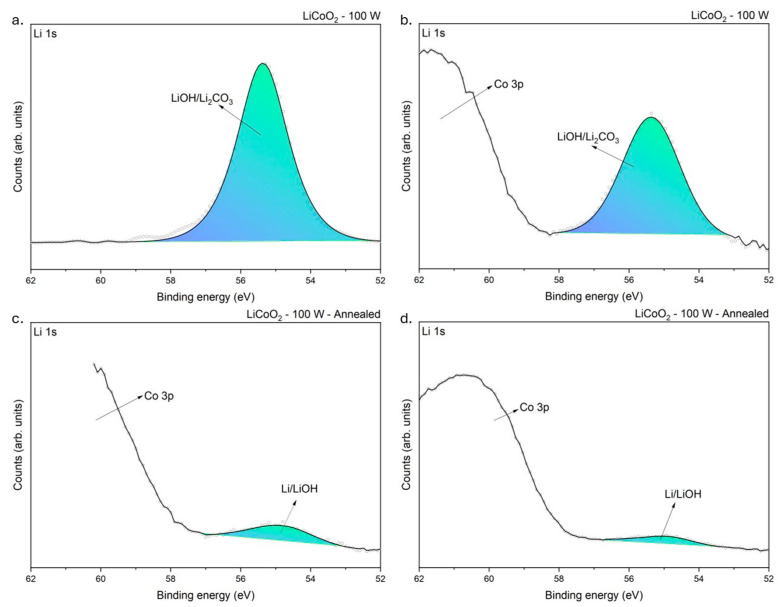
XPS spectra of LiCoO_2_ coatings before (**a**,**c**) and after (**b**,**d**) 200 s of Ar^+^ bombardment at a Li 1s binding level.

Figure 8 shows the XPS spectra obtained for the Li 1s binding energy level. The untreated sample (Figure 8a) showed a single peak at approximately 55.37 eV. According to Moulder et al. [29], this peak likely corresponded with lithium hydroxide (LiOH) and lithium carbonate (Li_2_CO_3_). This observation was consistent with the O 1s core-level spectrum obtained from the same sample (Figure 7a,b). Indeed, Ghorbanzade, López-Aranguren, and López del Amo [30] reported that these compounds were commonly formed due to the exposure of Li-rich materials to atmospheric humidity. This indicated that the coating was highly hygroscopic and readily reacted with H_2_O and CO_2_ in the ambient environment [30]. The formation of hydroxides with reactive elements like Li is common upon exposure to air [31]. Lithium’s high chemical reactivity drives sequential reactions, beginning with LiOH formation and subsequently forming Li_2_CO_3_ [30]. This sequence aligns thermodynamically with the reported standard Gibbs free energy of formation values, where the formation of Li_2_CO_3_ (${\Delta G}_{f}^{º}$ = −1216 kJ/mol) is more spontaneous than that of LiOH (${\Delta G}_{f}^{º}$ = −485 kJ/mol) [35]. After etching (Figure 8b), the untreated sample revealed an additional peak corresponding with Co 3p binding energy [36], confirming that Co resided beneath a Li-rich layer, as evidenced by the EDS linear profiling (Figure 2).

Regarding the annealing effect, the XPS spectra of the annealed sample confirmed the presence of the Co 3p core-level signal within the Li 1s core-level energy range, consistent with the survey spectra results (Figure 5). The annealed sample displayed a peak at approximately 54.72 eV, where the binding energies of LiOH and pure Li overlap [29]. Although LiCoO_2_ was not explicitly detected in this spectrum, its presence could not entirely be excluded due to peaks observed in the C 1s core-level spectrum (similar to Figure 6). However, the significant masking effect of the Co 3p core level, combined with insufficient spectral resolution, prevented the clear differentiation of all compounds. Consequently, the peak was labeled as Li/LiOH due to the mentioned binding energy overlap.

**Figure 9:**
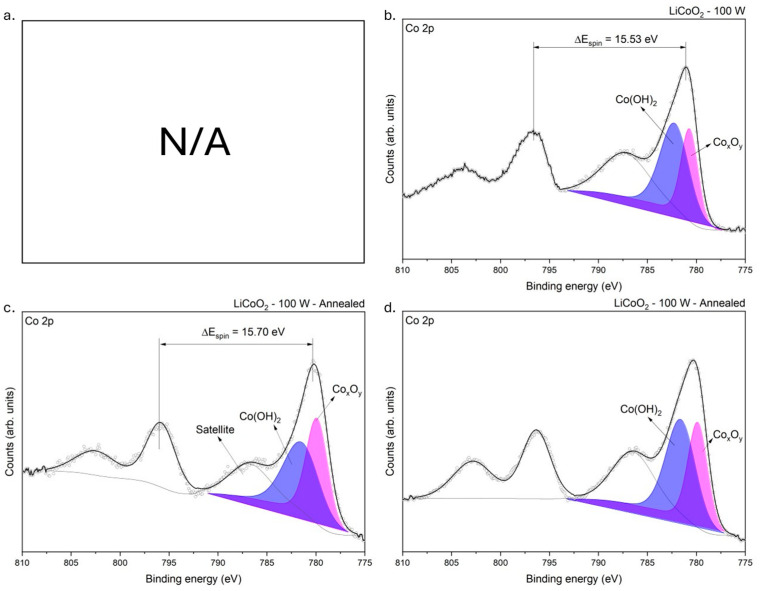
XPS spectra of LiCoO_2_ coatings before (**a**,**c**) and after (**b**,**d**) after 200 s of Ar^+^ bombardment at a Co 2p binding level.

**Figure 10:**
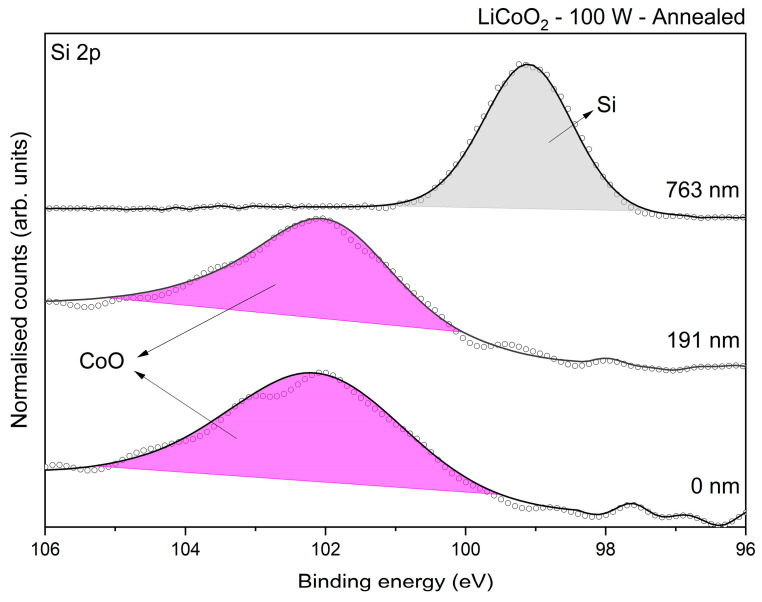
Si 2p core level of annealed LiCoO_2_ at different depths.

Figure 9 shows the Co 2p core-level spectrum obtained for the samples. Co was not detected in the as-deposited state, as illustrated in Figure 9a. This absence was likely due to lithium’s high reactivity, which promotes the formation of compounds such as LiOH and Li_2_CO_3_, pushing Li to diffuse toward the outermost layer and leaving Co concentrated in the inner region of the coating. The binding energy difference between the spin orbitals (ΔE_spin_) was approximately 15.6 eV, consistent with the values reported in the literature [29]. Regardless of the cleaning or treatment state, the coating exhibited two peaks at approximately 780.44 eV (labelled as Co_x_O_y_) and 781.88 eV. The first peak could represent a mixture of cobalt oxides, such as CoO, Co_3_O_4_, or Co_2_O_3_ [29]. However, according to Biesinger et al. [37], the overlapping binding energies of various cobalt oxides and hydroxides make the accurate quantification of these species difficult. The geometry of the Co 2p 3/2 spin level eliminated the possibility of metallic Co or Co_3_O_4_ being present [37]. The most likely explanation was a mixture of CoO, corresponding with the peak at ~780.44 eV, and Co(OH)_2_, associated with the peak at ~781.88 eV. In addition, the Co 3s core-level spectrum of the annealed sample showed a single consistent peak at ~102 eV, which corresponded with CoO species, as reported by McIntyre et al. [38] (see Figure 10).

Summarizing the XPS results, the analysis indicated that LiCoO_2_ decomposed after deposition due to its high reactivity with environmental components in the deposition and handling setup (e.g., unavoidable exposure to H_2_O and CO_2_ in ambient air, and residual gases in the deposition chamber and glovebox). LiOH was consistently present in the coatings, while Li_2_CO_3_ appeared to be present only in the untreated sample. However, the O 1s spectra revealed carbonate (CO_3_) bonds, suggesting that Li_2_CO_3_ could not entirely be ruled out after annealing. The redistribution of cobalt during annealing appeared to play a significant role in masking lithium species. Additionally, the XPS spectra confirmed that CoO was the sole cobalt oxide phase present in the coatings, regardless of their treatment or etching state, in agreement with the Raman results. Moreover, the spectra demonstrated that the deposited coating comprised a Li-rich outer layer and a Co-rich inner layer, which was consistent with the cross-sectional SEM micrograph and chemical linear profiling observed in Figure 2. After annealing, the coating exhibited a more uniform distribution of Li and Co, with an increased oxygen content.

### 3.2. Electrochemical Features of LiCoO_2_

#### 3.2.1. SEM Analysis

**Figure 11:**
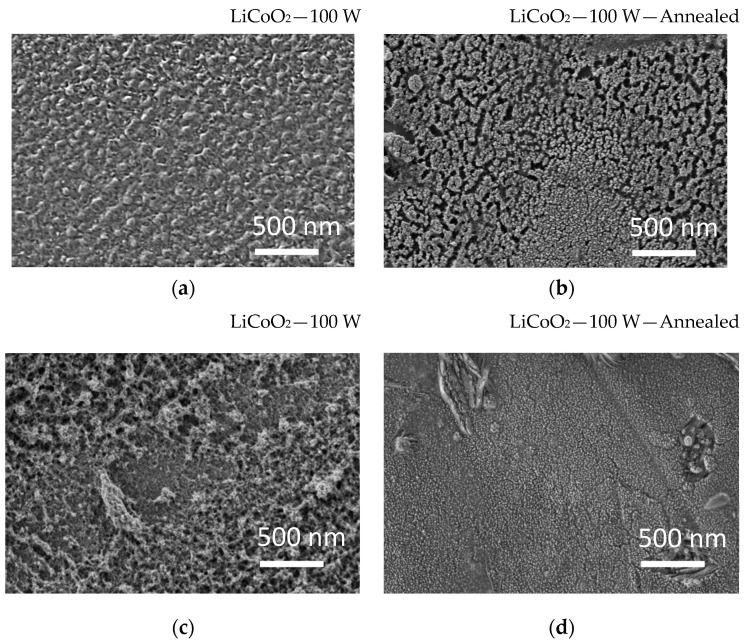
SEM images before (on the left) and after (on the right) annealing for the LiCoO_2_ coating deposited on glass substrates before (**a**,**b**) and after (**c**,**d**) 10 cycles.

Figure 11 shows the SEM images of the LiCoO_2_ coating before and after annealing and cycling. The annealing process showed the formation of cracks on the surface of the LiCoO_2_ coating (Figure 11a,b). Indeed, higher annealing temperatures (above 550 °C) can enhance crystallinity but induce stress and structural changes that lead to cracking [18,39]. After 10 cycles, the untreated LiCoO_2_ coating showed the formation of a porous top layer (Figure 11c), indicating the interaction between the electrolyte and the coating, while the annealed LiCoO_2_ coating (Figure 11d) revealed a denser top layer covering the formed cracks. Tan et al. [40] reported that prolonged cycling significantly degrades the LiCoO_2_ surface, resulting in structural damage at the electrode–electrolyte interface. This indicated that the layered LiCoO_2_ structure exhibited low stability near its surface when more than 60% of the lithium was extracted.

#### 3.2.2. CV and GCD Analyses

**Figure 12:**
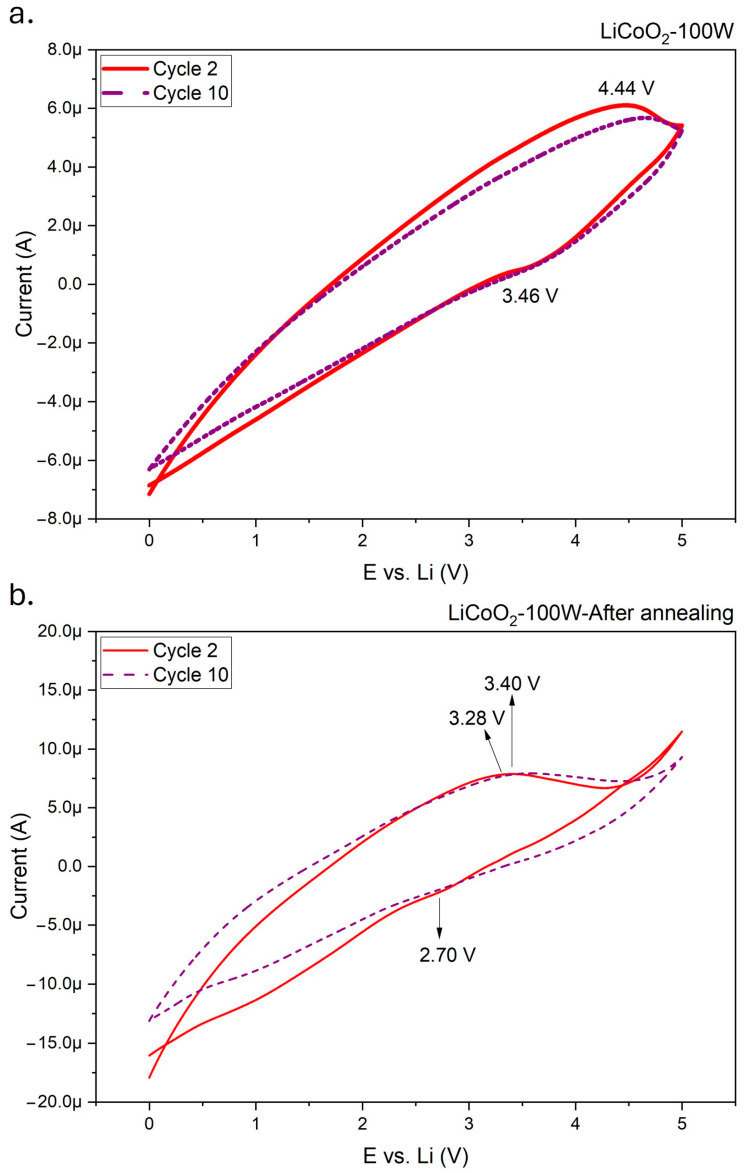
CVs for LiCoO_2_ coating before (**a**) and after (**b**) annealing in a 1 M LiClO_4_/EC:DEC solution. Scan rate: 1 mV/s.

Figure 12 presents the cyclic voltametric (CV) profiles of the LiCoO_2_ coating before and after annealing.

The unannealed sample exhibited a pair of broad, poorly defined anodic/cathodic peaks located at 4.44/3.46 V, indicating a single-step Li-ion insertion/deinsertion reaction and the associated redox process between Co^3+^ and Co^4+^ in the Li_x_CoO_2_ phase [41,42]. The unusual peak shape and the significant potential difference between the anodic and cathodic peaks may have resulted from the scan rate used during the CV measurements. At higher scan rates, peak overlapping can occur due to the rapid movement of ions, increasing the current and leading to greater irreversibility in the redox process, as reflected by widened anodic and cathodic peak potentials [43]. After annealing, the cyclic voltammetry results showed a significant increase in the capacity of the LiCoO_2_ coating, as evidenced by a higher current range on the Y-axis of Figure 12. However, the redox process occurred at lower potentials, with peaks detected at 3.28/2.70 V. This suggested that annealing reduced the potential required for Li-ion insertion/extraction in the LiCoO_2_ coating. This behavior was likely influenced by the presence of additional crystallographic planes, as observed in the XRD results. Recrystallization following annealing likely enhanced Li-ion diffusion, reducing the potential needed for the reaction. Regardless of the coating’s state (annealed or unannealed), the potential required to trigger the redox reaction increased during the charging portion of the hysteresis curve in the CVs. Conversely, the potential required to trigger the reaction decreased during the discharging portion of the curve. These slight variations could be associated with the degradation of the coating’s electrochemical properties, a common phenomenon after repeated cycling [43].

Based on the XPS results, Co species in the LiCoO_2_ coating (e.g., CoO and Co(OH)_2_) may have transformed into Co_3_O_4_ as the potential increased. This transformation was a clear indication of lithium removal from the coating, which promoted the oxidation of cobalt to the Co^4+^ ionic state [44]. After annealing, the coating remained in the Co^2+^ zone, as suggested by the potentials recorded in the CVs. This implied that the coating may have undergone continuous oxidation or hydration, similar to the behavior observed in Figure 9 under this operating regime.

**Figure 13:**
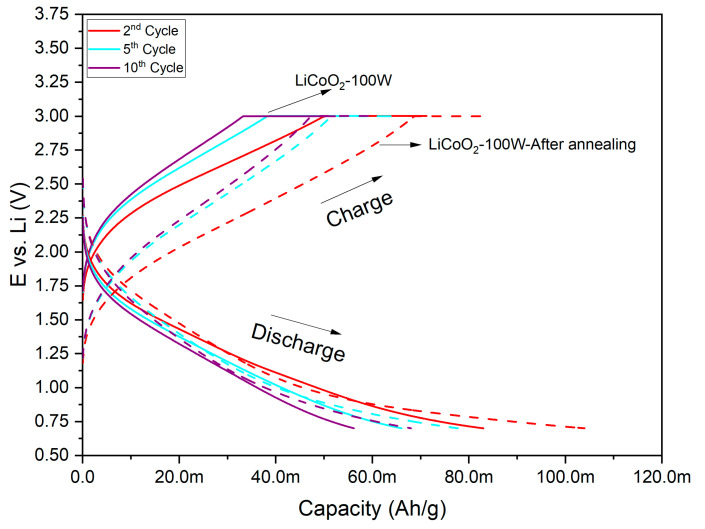
Charge/discharge curves of the coating before (solid line) and after (dashed line) annealing at a 0.85 C rate.

The charge/discharge plots illustrated the gradual degradation of the coating, as evidenced by the reduction in capacity with an increasing number of cycles (Figure 13). As discussed in the CV results, annealing the LiCoO_2_ coating significantly enhanced its capacity (see values on the X-axis), representing a notable improvement for practical applications. Comparing the discharge capacities at 0.7 V after 10 cycles, the annealed coating retained approximately 65% of its discharge capacity, while the unannealed sample retained about 68%. It is noteworthy that the discharge curves did not exhibit the typical potential drop, maintaining a specific capacity as expected. This behavior could be associated with the applied rate (0.85 C) during the measurements, suggesting partial lithiation/delithiation during the charge/discharge cycling. In terms of charge capacity retention, comparable values were observed after 10 cycles (~71% for the annealed coating and ~73% for the unannealed sample). This rapid degradation was expected under the tested conditions. The annealing process appeared to have had a minimal effect on capacity retention, consistent with Figure 13, which shows a larger specific capacity difference between the second and the tenth cycle in the annealed sample compared with the unannealed one. The first charge/discharge cycle was excluded from the measurements due to the inability to ensure identical initial charge conditions across samples.

**Figure 14:**
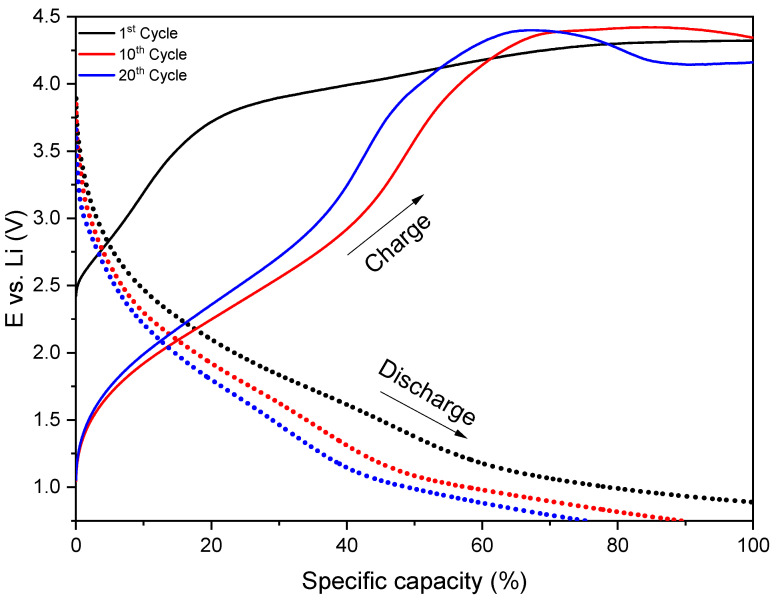
Charge (solid line)/discharge (dashed line) curves of the annealed LiCoO_2_ at a 0.85 C rate with potentials from 0.7 to 4.5 V.

An additional galvanostatic charge/discharge test was performed on the annealed sample, with the results displayed in Figure 14. As previously mentioned, the absence of a potential drop during the test indicated partial lithiation/delithiation in the coating. The higher applied potential during charging accelerated the degradation process, as expected. With an increase in the number of cycles, the charge curve underwent significant changes. After 10 cycles, a plateau became evident, which transformed into a broad peak after 20 cycles. This likely reflected the effects of coating degradation under this testing regime, significantly impacting discharge capacity retention, which decreased by approximately 13%. Another potential effect of the higher charging potential was the formation of oxygen-rich compounds, such as LiO_3_, or an increased concentration of Co_3_O_4_ in the coating, as suggested by the XPS spectra (Figure 9). These observations highlighted the complex degradation mechanisms occurring in the coating under elevated potentials.

## 4. Conclusions

LiCoO_2_ thin films were deposited at 100 W using RF magnetron sputtering in an argon atmosphere. Post-deposition analyses, including XRD, Raman, and EDS, revealed the formation of a Li_2_CO_3_ surface layer due to air exposure. The XPS analysis substantiated these findings, detecting lithium in the form of carbonate (Li_2_CO_3_) and hydroxide (LiOH) species. Additionally, CoO was observed after etching, supporting the results from the XRD, Raman, and EDS analyses. Subsequent annealing at 600 °C for 1 h in an argon atmosphere significantly enhanced the crystallinity of the LiCoO_2_ coating compared with the as-deposited film. Raman spectroscopy indicated the partial removal of the Li_2_CO_3_ surface layer, although the XPS analysis revealed persistent carbonate bonds in the O 1s spectra, suggesting that the complete elimination of Li_2_CO_3_ was likely not achieved. CoO was confirmed as the sole cobalt oxide phase post-annealing, consistent with the Raman findings. XPS and SEM chemical profiling further revealed a layered structure in the as-deposited film, with a Li-rich outer layer and a Co-rich underlayer, whereas annealing promoted a more uniform distribution of Li and Co, accompanied by increased oxygen content. To evaluate the effect of annealing on the performance of LiCoO_2_, including its capacity, cycling stability, and rate capability, CV and GCD analyses were conducted. Electrochemical testing demonstrated that the annealed LiCoO_2_ coating exhibited a higher capacity than the unannealed sample. However, after 10 cycles, the annealed coating retained approximately 65% of its discharge capacity, while the unannealed sample retained about 68%. The formation of a denser top layer post-cycling in the annealed coating suggested an improved interaction with the electrolyte, despite the presence of cracks. These results elucidated how the annealed film’s enhanced chemical structure positively influenced its electrochemical performance as a cathode. Collectively, these findings highlight the potential of annealed LiCoO_2_ thin films as cathode materials for lithium-ion batteries.

## Figures and Tables

**Table 1:** XPS peak parameters of LiCoO_2_ coatings.

Phase/Compound	Peak	Binding Energy—E_b_ (eV)	FWHM (eV)
C−O−H	C 1s	285.94 ± 0.00	2.68 ± 0.00
C−O_3_	C 1s	289.83 ± 0.36	2.28 ± 0.40
O	O 1s	529.67 ± 0.27	1.50 ± 0.46
CoO	O 1s	530.26 ± 0.00	1.57 ± 0.00
CO_3_/OH	O 1s	531.73 ± 0.10	1.99 ± 0.42
Li/LiOH	Li 1s	54.72 ± 0.02	1.88 ± 0.22
LiOH/Li_2_CO_3_	Li 1s	55.37 ± 0.01	1.82 ± 0.12
Co_x_O_y_	Co 2p	780.44 ± 0.20	2.84 ± 0.57
Co(OH)_2_	Co 2p	781.88 ± 0.32	3.50 ± 0.04

## Data Availability

The original contributions presented in this study are included in the article. Further inquiries can be directed to the corresponding author.
